# 2-Methoxyestradiol Damages DNA in Glioblastoma Cells by Regulating nNOS and Heat Shock Proteins

**DOI:** 10.3390/antiox11102013

**Published:** 2022-10-12

**Authors:** Paulina Emilia Bastian, Agnieszka Daca, Agata Płoska, Alicja Kuban-Jankowska, Leszek Kalinowski, Magdalena Gorska-Ponikowska

**Affiliations:** 1 Department of Medical Chemistry, Medical University of Gdansk, 80-210 Gdansk, Poland; 2 Department of Pathology and Experimental Rheumatology, Medical University of Gdansk, 80-210 Gdansk, Poland; 3 Department of Medical Laboratory Diagnostics—Fahrenheit Biobank BBMRI.pl, Faculty of Pharmacy, Medical University of Gdansk, 80-211 Gdansk, Poland; 4 BioTechMed Centre, Department of Mechanics of Materials and Structures, Gdansk University of Technology, Narutowicza Street 11/12, 80-233 Gdansk, Poland; 5 Department of Biophysics, Institute of Biomaterials and Biomolecular Systems, University of Stuttgart, D-70569 Stuttgart, Germany; 6 Euro-Mediterranean Institute of Science and Technology, 90139 Palermo, Italy

**Keywords:** 2-methoxyestradiol, glioblastoma, oxidative stress, reactive nitrogen species, nitric oxide synthase, heat shock protein

## Abstract

Gliomas are the most prevalent primary tumors of the central nervous system (CNS), accounting for over fifty percent of all primary intracranial neoplasms. Glioblastoma (GBM) is the most prevalent form of malignant glioma and is often incurable. The main distinguishing trait of GBM is the presence of hypoxic regions accompanied by enhanced angiogenesis. 2-Methoxyestradiol (2-ME) is a well-established antiangiogenic and antiproliferative drug. In current clinical studies, 2-ME, known as Panzem, was examined for breast, ovarian, prostate, and multiple myeloma. The SW1088 grade III glioma cell line was treated with pharmacological and physiological doses of 2-ME. The induction of apoptosis and necrosis, oxidative stress, cell cycle arrest, and mitochondrial membrane potential were established by flow cytometry. Confocal microscopy was used to detect DNA damage. The Western blot technique determined the level of nitric oxide synthase and heat shock proteins. Here, for the first time, 2-ME is shown to induce nitro-oxidative stress with the concomitant modulation of heat shock proteins (HSPs) in the SW1088 grade III glioma cell line. Crucial therapeutic strategies for GMB should address both cell proliferation and angiogenesis, and due to the above, 2-ME seems to be a perfect candidate for GBM therapy.

## 1. Introduction

### 1.1. Glioma

Gliomas are the most prevalent kind of primary central nervous system cancer (CNS). They come from astrocytes and oligodendrocytes, which are collectively referred to as glial cells. Gliomas are most prevalent in the brain, although they may affect the whole CNS [1]. According to the World Health Organization (WHO) classification from 2007, the primary glial tumor categories include astrocytic, oligodendroglial, oligoastrocytic, ependymal, neuronal, and mixed neuronal–glial tumors (such as gangliogliomas) [2]. In this classification, histologic features and grading based on degrees of malignancy are crucial for diagnosis and treatment. Tumors are categorized from WHO grade I to grade IV, primarily based on increasing malignancy, including the presence and degree of atypia and mitotic activity. Particular hallmarks for certain subtypes include microvascular proliferation and ‘pseudopalisading’ necrosis in grade IV glioblastoma multiforme (GBM) [3]. Low-grade gliomas (LGGs) often consist of grade I and grade II gliomas. Grade I gliomas have a modest proliferation capacity, are largely circumscribed, and are curable with surgical resection. Grade II gliomas are often invasive growths that exhibit minimal proliferative activity as well as a low level of malignancy. Nevertheless, if the extent of resection is inadequate, remaining lesions will return and even grow into high-grade lesions, thereby threatening the patient’s life [4,5,6]. In addition, although the surgical prognosis for individuals with an LGG is favorable, malignant transformation occurs in certain patients, which later refers to the evolution of an LGG to a high-grade glioma (HGG) (WHO grade III or grade IV tumor) [7,8]. The majority of HGG juvenile CNS glial tumors are anaplastic astrocytomas (AA, WHO grade III) and glioblastomas (GBM, WHO grade IV) [9]. In children, HGGs are most frequently detected in the primary care system, but as in adults, they can also emerge from the transition of an LGG [10].

Gliomas of astrocytic origin have a minimal response to treatment. Chemoresistance is especially prominent in GMB, the most prevalent and aggressive kind of glioma. The failure of chemotherapy is partially due to mechanisms against frequently used DNA-alkylating chemicals but also to the constitutive activation of the pro-survival phosphatidylinositol 3-kinase (PI3K)–protein kinase B (Akt) pathway, which suppresses apoptosis, in glioma cells. Therefore, novel therapeutics with a mechanism distinct from DNA alkylation are necessary [1]. Moreover, a high level of vascular endothelial growth factor (VEGF) expression in GMB tumor tissues is a significant indication of a poor therapeutic response. This brings us to the conclusion that antiangiogenic drugs are the most promising novel treatments for GBM [11].

HGG patients have a very dismal prognosis. Resistance to traditional treatment approaches and the presence of a heterologous cell population, including astrocytic and vascular features that promote pathological angiogenesis, are the primary obstacles. Important therapeutic approaches must target both cell growth and angiogenesis. 2-Methoxyestradiol (2-ME), a physiologic estradiol derivative and a well-known antiangiogenic and anticancer drug, seems to be an ideal option for HGG treatment [12].

### 1.2. 2-Methoxyestradiol—Natural Metabolite and Its Anticancer Activity

2-Methoxyestradiol (2-ME) is a metabolic byproduct of 17β-estradiol (E2). 2-ME synthesis in the organism involves two steps: first, the hydroxylation of E2 to 2-hydroxyestradiol by an NADPH-dependent cytochrome P-450-linked monooxygenase system, followed by the O-methylation of 2-hydroxyestradiol, facilitated by catechol-O-methyltransferase (COMT), resulting in the formation of monomethyl ether—2-ME [13]. Serum concentrations of 2-ME vary from 30 pM in males to 50 nM in pregnant women; nevertheless, pharmacological doses (1–10 μM) of 2-ME have shown anticancer and antiangiogenic effects in experimental models of cancer [14,15]. The anticancer effect of 2-ME is closely linked to the development of nitro-oxidative stress that leads to the apoptosis of proliferating and cancerous cells [12]. Panzem is being studied in current clinical studies for ovarian, breast, prostate, and multiple myeloma cancer therapy [16,17,18]. The molecular mechanism of 2-ME still remains unclear; however, Gorska et al. demonstrated that 2-ME enhanced the nuclear fraction of neuronal nitric oxide synthase (nNOS) in a pediatric osteosarcoma cell type (143b) [19,20], resulting in the release of nitric oxide (NO) molecules. This elevated NO level results in DNA strand breakage and, ultimately, cell death [21,22]. Moreover, pharmacological doses of 2-ME trigger apoptosis in mouse hippocampus HT22 cells, which poses the question of whether 2-ME may lead to neurodegenerative disorders and act as a hormone per se [23,24,25]. Furthermore, 2-ME was demonstrated to regulate mitochondrial biogenesis in 143b cells, particularly at physiologically relevant doses. As a consequence of the nuclear recruitment of nNOS and NO production, it suppresses mitochondrial biogenesis through the modulation of peroxisome proliferator-activated receptor gamma coactivator (PGC)-1, cyclooxygenase-1 (COX-1), and sirtuin 3 (SIRT3) [26].

According to the authors’ knowledge, the 2-ME influence on the SW1088 grade III glioma cell line was never examined before. In this article, the results are compared to the examination of other gliomas and other cancer cell lines described before. Due to the above, this research article presents, for the first time, the induction of nitro-oxidative stress with the simultaneous regulation of heat shock proteins (HSPs) in the SW1088 grade III glioma cell line by 2-ME at both pharmacological and physiological concentrations.

## 2. Materials and Methods

### 2.1. Cell Line and Cell Culture

The human SW1088 grade III glioma ([SW-1088, SW 1088] (ATCC^®^ HTB12™) cell line was supplied by ATCC (American Type Culture Collection, HTB-12, Manassas, VA, USA). The cells were cultured at 37 °C in a humidified atmosphere saturated with 5% CO_2_ using Dulbecco’s Modified Eagle’s Medium with high glucose (D5796, Sigma Aldrich, Poznań, Poland) supplemented with 2 mM glutamine (59202C Sigma Aldrich, Poland), 1% non-essential amino acids (M7145, Sigma Aldrich, Poland), 10% fetal bovine serum (F7524, Sigma Aldrich, Poland), and a penicillin (100 μg/mL)/streptomycin (100 μg/mL) cocktail (P4333, Sigma Aldrich, Poland).

### 2.2. Cell Line Treatment

To investigate the influence of physiological and pharmacological concentrations of 2-ME, the cells were treated with physiological (100 pM, 1 nM, and 10 nM) and pharmacological (100 nM, 1 µM, and 10 µM) concentrations of 2-ME (M6383, Sigma Aldrich, Poland) for 24 h. To eliminate the impact of hormones obtained from sera, all studies were conducted on a medium devoid of fetal bovine serum. Control cells were treated with an identical proportion of the solvent used to create 2-ME solutions, DMSO (dimethyl sulfoxide, D2438, Sigma Aldrich, Poland). The final concentration of DMSO in the medium for incubation was below 0.1%.

### 2.3. Cell Viability Test (MTT Test)

SW1088 cells were seeded at a density of 10,000 per well in a 96-well plate. After 24 h, the cell culture medium was discarded, and the cells were treated with 2-ME in a concentration range of 100 pM–10 µM for another 24 h. Cells treated with the solvent were the control (100 percent cell viability). At a concentration of 0.5 mg/mL, 3-[4,5-dimethylthiazol-2-yl]-2,5-diphenyltetrazolium bromide (MTT) was added after the appropriate incubation period (M2128, Sigma-Aldrich, Poznań, Poland). After incubating the plates at 37 °C for four hours, the supernatant was removed by centrifugation (700× *g* for 10 min). Lastly, 100 μL of DMSO was added to dissolve the formazan crystals. Absorbance at 540 nm was read using a microplate reader (BioTek Instruments, Inc., Winooski, VT, USA). The data are presented as a percentage of the control. Each experiment was repeated a minimum of three times.

### 2.4. Flow Cytometric Analysis of Apoptosis and Necrosis

Flow cytometry was used to evaluate the levels of apoptosis and necrosis. SW1088 cells were seeded in 6-well plates at a density of 300,000 cells per well. After 24 h, the cells were treated with 2-ME in a concentration range of 100 pM–10 µM for an additional 24 h. Following trypsinization, the cells were collected by centrifugation at 1200× *g* for 7 min and washed 3 times with ice-cold phosphate buffer saline (PBS, 137 mM NaCl, 2.7 mM KCl, and 4.3 mM Na_2_HPO_4_, pH 7.4). The cells were then incubated for 15 min at room temperature with annexin V and PI (propidium iodide) (559763, PE Annexin V Apoptosis Detection Kit I, BD Biosciences, Berkshire, England). The procedure was carried out on ice, except for incubation with annexin V and PI. On a BD FACSVerse flow cytometer (Becton-Dickinson, Franklin Lakes, NJ, USA), the fluorescence signals of annexin V and PI conjugate were detected in fluorescence intensity channels FL1 and FL3, respectively. The results were analyzed using FlowJo software, version 10.6.1 (FlowJo LCC, Becton Dickinson, Ashland, OR, USA). At least three repetitions of the process were conducted to confirm the repeatability of the results.

### 2.5. Flow Cytometry Analysis of the Cell Cycle

Flow cytometry was utilized to analyze the cell cycle. SW1088 cells were seeded in 6-well plates at a density of 300,000 cells per well. After 24 h, the cells were treated with 2-ME in a concentration range of 100 pM–10 µM for an additional 24 h. Following trypsinization, the cells were collected by centrifugation at 1200× *g* for 7 min. The samples were rinsed with ice-cold PBS and then fixed overnight at 4 °C with ice-cold 70% ethanol. The cells were then centrifuged for 7 min at 1200× *g*. The cells were then treated with 5 µg of RNase A (E1350-02, EURX, Gdańsk, Poland) to stain the DNA. The final stage involved adding 10 µg of PI (51-66211E, BD Biosciences). The results were analyzed with FlowJo software version 10.6.1. At least three repetitions of the process were conducted to confirm the reproducibility of the results.

### 2.6. Flow Cytometric Analysis of Reactive Oxygen Species (ROS)

SW1088 cells were seeded at a density of 300,000 cells per well in 6-well plates. Next, the cells were incubated with 2-ME in a concentration range of 100 pM–10 µM for 8 h. The levels of ROS were measured by the fluorescence intensity of 2′,7′-dichlorofluorescein diacetate (DCF DA, D6883, Sigma-Aldrich, Poland), which was added at a final concentration of 10 μM 30 min prior to the end of the incubation period. Trypsin was used to detach the cells from the plates before they were collected and centrifuged (1200× *g* for 5 min). The cells were washed twice with PBS, suspended in PBS, and then analyzed by flow cytometry. The entire operation was performed on ice. A total of 30,000 cells were counted and analyzed using flow cytometry (BD FACSVerse). The results were analyzed with FlowJo software version 10.6.1. At least three repetitions of the procedure were conducted to ensure the repeatability of the results.

### 2.7. Flow Cytometric Analysis of Reactive Nitrogen Species (RNS)

SW1088 cells were seeded at a density of 300,000 cells per well in 6-well plates. Next, the cells were incubated with 2-ME in a concentration range of 100 pM–10 µM for 8 h. The level of RNS was measured by the fluorescence intensity of 4-amino-5-methylamino-2’,7’-difluorescein diacetate (DAF-FM DA, D2321, Sigma-Aldrich, Poland), which was added at a final concentration of 10 μM 30 min prior to the end of the incubation period. Trypsin was used to detach the cells from the plates before they were collected and centrifuged (1200× *g* for 5 min). The cells were washed twice with PBS, suspended in PBS, and then analyzed by flow cytometry. The entire operation was performed on ice. A total of 30,000 cells were counted and analyzed using flow cytometry (BD FACSVerse). The results were analyzed with FlowJo software version 10.6.1. At least three repetitions of the procedure were conducted to ensure the repeatability of the results.

### 2.8. Analysis of Mitochondrial Potential by Flow Cytometry

The mitochondrial membrane potential was investigated based on the aggregation reaction of the lipophilic cationic dye 5.5′,6.6′-tetrachloro-1.1′,3.3′-tetraethylbenzimidazolocarbocyanine iodide (JC-1, Cayman). The selective accumulation of JC-1 in mitochondria depends on the mitochondrial potential (ΔΨm). In the case of high ΔΨm, the JC-1 dye forms aggregates that emit red fluorescence (ex/em 535/595 nm). When the mitochondrial membrane is depolarized (ΔΨm decrease), this dye, due to its translocation into the cytoplasm, turns into the monomeric form, emitting green fluorescence (ex/em 485/535 nm). These features make JC-1 a sensitive marker of mitochondrial membrane potential changes, and measuring the aggregate-to-monomer ratio of fluorescence is a convenient and reliable way to assess mitochondrial membrane potential changes in cells [27]. The ratio between the fluorescence of aggregates (λem = 595 nm) and monomers (λem = 535 nm) reflects the level of damage to the mitochondrial membranes of cells.

The SW1088 cells were seeded at a density of 300,000 cells per well in 6-well plates. After 24 h, cells were treated with 2-ME in a concentration range of 100 pM–10 µM for 24 h. The 2 mM JC-1 solution was added and incubated for 30 min at 37 °C. Cells were then trypsinized and harvested by centrifugation at 1200× *g* for 7 min. The cells were further washed twice with PBS and centrifuged at 850× *g* for 5 min. Cells treated with 50 µM CCCP (carbonyl cyanide m-chlorophenyl hydrazone) for 15 min were the positive control, as it inhibits oxidative phosphorylation by the uncoupling of the proton gradient and thus causes ATP synthase inhibition, leading to a reduction in the mitochondrial membrane potential. The analysis of the level of the mitochondrial membrane potential was performed on a FACSVerse flow cytometer (Becton Dickinson). The results were analyzed with FlowJo software version 10.6.1. At least three repetitions of the procedure were conducted to ensure the repeatability of the results.

### 2.9. Western Blot Analysis

The level of neuronal nitric oxide synthase (nNOS, ab5583, Abcam, Cambridge, UK), endothelial nitric oxide synthase (eNOS, ab66127, Abcam, UK), inducible nitric oxide synthase (iNOS, ab15323, Abcam, UK), heat shock protein 70 (Hsp70, ab45133, Abcam, UK), heat shock protein 60 (HSP60, sc-13115, Santa Cruz, CA, USA), heat shock protein 90 (HSP90, ab80159, Abcam, UK) proteins and β-actin (A3854, Sigma, Poland) were determined using the Western blot method. The cells were seeded on cell culture plates and, after 24 h at 80% confluence, were treated with 2-ME in a concentration range of 100 pM–10 µM for the next 24 h. The cells were then scraped out and centrifuged. The pellets were suspended in RIPA buffer (R0278, Sigma-Aldrich, Poland) and a mixture of protease inhibitors after being washed three times with PBS (Sigma-Aldrich, Poland). The Bradford reagent (Sigma-Aldrich, Poland) was used to calculate the protein concentration. After that, samples containing 20 µg of protein were combined with Laemmli loading buffer (Sigma-Aldrich, Poland) and incubated at 95 °C for 5 min. Electrophoresis was used to separate the proteins on 4–20% Mini-PROTEAN^®^ TGX Stain-Free^TM^ Protein Gels (4568093, Bio-rad, Hercules, CA, USA) (120 mV, 90 min). Using the Trans-Blot Turbo System, the separated proteins were transferred to Trans-Blot Turbo Midi 0.2 µm PVDF (1704157, Bio-rad, USA). The membranes were then treated with primary antibodies overnight at 4 °C after being blocked for 45 min in 5% nonfat milk in TBS-T (0.5% Tween20, 20 mM Tris-HCl, pH 7.4, and 0.5 M NaCl). After being washed 3 times for 10 min in TBS-T, the membranes were incubated with horseradish peroxidase (HRP)-conjugated secondary antibodies (1:50,000 dilution in TBS-T) for 1 h at room temperature (Anti-rabbit IgG peroxidase antibody produced in goat, A0545, Sigma Aldrich, Goat Anti-Mouse IgG HRP, 10004302, Cayman Chemical Company). Later, the membranes were washed three times for ten minutes each time in TBS-T. According to the manufacturer’s instructions, visualization was carried out using chemiluminescence enhanced with a Luminata^TM^ Crescendo Western HRP Substrate (Millipore Corporation, Burlington, MA, USA). The ImageQuant LAS 500 was used to read the signal (GE Healthcare, Kraków, Poland). Using densitometry analysis by Quantity One 4.6.8, protein levels were calculated. The results were adjusted to β-actin. At least three different repetitions of each experiment were performed.

### 2.10. DNA Strand Breaks Observed by Confocal Microscopy

DNA breakage within the nucleus is a characteristic of apoptosis. Using the dUTP end-labeling (TUNEL, Terminal deoxynucleotidyl transferase-mediated d-UTP Nick End-Labeling) method mediated by terminal deoxynucleotidyl transferase (TdT), DNA cleavage in apoptotic cells can be identified in situ in fixed cells. The TUNEL assay is a very specific method for detecting apoptotic cells. The TdT enzyme catalyzes the addition of labeled dUTP to the 3’ ends of cleaved DNA fragments in the TUNEL assay. Digoxigenin-dUTP or biotin-dUTP can be identified using secondary reagents (such as anti-digoxigenin or streptavidin antibodies) for fluorescence or colorimetric detection. Alternately, dUTP coupled to a fluorescent dye can be utilized for the direct detection of fragmented DNA using confocal microscopy.

SW1088 cells were plated at a density of 300,000 cells per well onto round glass coverslips placed in 6-well plates. After 24 h, cells were treated with 2-ME in a concentration range of 100 pM–10 µM for 24 h. The TUNEL assay was carried out using the Tunel Andy Fluor^TM^ 488 Apoptosis Detection Kit (A050, ABP Biosciences, Beltsville, MD, USA) following the manufacturer’s instructions. Briefly, cells were fixed with 4% formaldehyde in PBS (pH 7.4) for 30 min at 4 °C, washed with PBS, and permeabilized with Triton X-100 (0.2% in PBS) for 30 min at room temperature. In the next step, coverslips were incubated in a humid chamber with a mixture containing the TdT enzyme and biotin-tagged- dUTP for 60 min at 37 °C, protected from light. In the final step, cells were incubated with Andy Fluor™ 488-Streptavidin staining solution for 30 min at room temperature, protected from light. Finally, coverslips were mounted onto microscopic slides with a mounting medium containing DAPI as a counterstain and left to dry. Using a confocal microscope (Opera PhenixTM, Perkin-Elmer, Waltham, MA, USA), digitized cell pictures were acquired the following day. Image analysis and merging were performed using Harmony (Perkin-Elmer, MA, USA) and ImageJ (v1.52a, NIH, Bethesda, MD, USA) software. The data are reported as relative fluorescence units (RFU) normalized to the control ratio. The ratio of relative fluorescence units (RFU) of the TUNEL-positive deoxyuridine triphosphate deoxyuridine transferase-positive cells is used to represent the data.

### 2.11. Statistical Analysis of Test Results Obtained

The results are the mean standard deviation of at least three separate experiments. The differences between control samples and 2-ME-treated samples were evaluated using one-way analysis of variance (ANOVA), followed by Dunnett’s multiple comparison test for post hoc analysis. A *p*-value of less than 0.01 was regarded as indicating statistical significance. Using GraphPad Prism, the data were examined (GraphPad Software, Inc., version 8, San Diego, CA, USA).

## 3. Results

### 3.1. 2-ME Inhibits Cell Viability

Aiming at elucidating the mechanisms of action of 2-methoxyestradiol, SW1088 grade III glioma cells were used in a series of experiments that began with the determination of dose cytotoxicity corresponding to the pharmacological and physiological concentrations of 2-ME.

**Figure 1:**
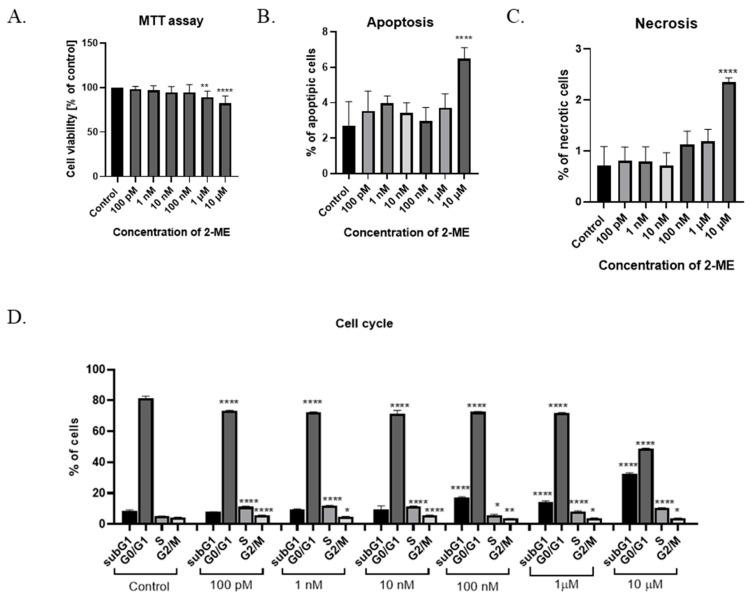
(**A**) Cell viability assay. (**B**) Induction of apoptosis by 2-ME. (**C**) Induction of necrosis by 2-ME. (**D**) Cell cycle arrest by 2-ME. Total cell levels in apoptosis and necrosis after 24 h incubation of SW1088 cells with 2-ME in a concentration range from 100 pM to 10 µM. Values are mean ± SE of three independent experiments. Data were analyzed with GraphPad Prism Software version 8.0.1 using bidirectional ANOVA with Dunnett’s multiple comparison test; * *p* < 0.05, ** *p* < 0.01, **** *p* < 0.0001 versus control.

The cytotoxicity of 2-ME was determined after the treatment of SW1088 grade III glioma cells, which, after 24 h incubation at pharmacological (10 µM, 1 µM, and 100 nM) and physiological (10 nM, 1 nM, and 100 pM) concentrations of this compound, were able to proliferate. Cell viability was determined by a microplate MTT spectrophotometric method. The percentage of viable cells in the sample was calculated in comparison to control cells assumed to be 100% viable. For grade III glioma cells, inhibited SW1088 cell growth was observed when incubated with 10 µM and 1 µM 2-ME, with 82% (±8.21) and 89% (±7.02) metabolically active cells, respectively. In the case of the remaining concentrations of 2-ME, no statistically significant effect was observed (Figure 1A).

### 3.2. Induction of Cell Death by 2-ME

To assess whether 2-ME induces a cytotoxic effect by inducing apoptosis and/or necrosis, SW1088 glioma cells were treated with 2-ME in a concentration range from 100 pM to 10 µM for 24 h; then, using flow cytometry, the levels of apoptosis and necrosis were assessed using the dye annexin V and propidium iodide (PI). The number of apoptotic cells significantly increased to 6.48% (±0.63) for samples treated with 10 µM 2-ME in comparison to the control, in which 2.69% (±1.38) underwent apoptosis (Figure 1B). Moreover, 10 µM 2-ME also induced necrosis in 2.35% (±0.08) in comparison to the control (0.71% (±0.37) (Figure 1C).

### 3.3. 2-ME Blocks Cell Cycle

In the next stage of the research, the influence of 2-ME on the cell cycle was assessed. The cells of the SW1088 grade III glioma cell line were treated with concentrations corresponding to the pharmacological (10 µM, 1 µM, and 100 nM) and physiological (10 nM, 1 nM, and 100 pM) concentrations of 2-ME for 24 h (Figure 1D). Subsequently, the distribution of cells in individual phases of the cell cycle was determined by flow cytometry using the propidium iodide dye. In the differentiated glioblastoma cell line SW1088, statistically significant changes in the cell cycle were observed at both the pharmacological and physiological concentrations of 2-ME (Figure 1D). The number of cells in the subG1 phase was increased after treating cells with pharmacological concentrations of 10 µM, 1 µM, and 100 nM 2-ME, and the results were 32.67 ± 0.46, 14.37 ± 0.57, and 16.97 ± 0.60, respectively relative to the control (8.93 ± 0.69). In the G0/G1 phase, a statistically significant decrease in the number of SW1088 cells was noted after the use of all concentrations of 2-ME, which were as follows: 10 μM—48.7 ± 0.37; 1 μM—71.87 ± 0.30; 100 nM—72.57 ± 0.23; 10 nM—71.50 ± 1.65; 1 nM—72.20 ± 0.29; and 100 pM—73.40 ± 0.16 compared to the control (81.63 ± 0.92). In the S phase, a statistically significant increase in the number of SW1088 cells was observed after the use of all concentrations of 2-ME, which were as follows: 10 µM—10.57 ± 0.04; 1 µM—7.97 ± 0.56; 100 nM—5.71 ± 0.56; 10 nM—11.53 ± 0.17; 1 nM—11.90 ± 0.14; and 100 pM—11.23 ± 0.29 versus the control (5.02 ± 0.16). In the G2/M phase, a statistically significant decrease in the number of SW1088 cells was observed after the use of pharmacological concentrations of 2-ME, which were: 10 µM—3.79 ± 0.13; 1 µM—3.79 ± 0.19; and 100 nM—3.60 ± 0.09, while in the case of physiological concentrations, the number of cells increased and amounted to 10 nM—5.54 ± 0.22; 1 nM—4.84 ± 0.17; and 100 pM—5.66 ± 0.10 versus the control (4.30 ± 0.19).

### 3.4. 2-ME Induces DNA Strand Breaks

The next step in the research was to check whether the nucleus could be targeted by treatment with 2-ME. The analysis of DNA fragmentation was performed using the TUNEL method. The TUNEL method allows the detection of apoptotic cells based on DNA fragmentation labeling. The use of terminal deoxynucleotide transferase (TdT) enables the attachment of FITC-labeled deoxyuridine triphosphates (FITC-dUTP) to the free 3′ ends of single- or double-stranded DNA breaks and direct analysis using fluorescence microscopy.

**Figure 2:**
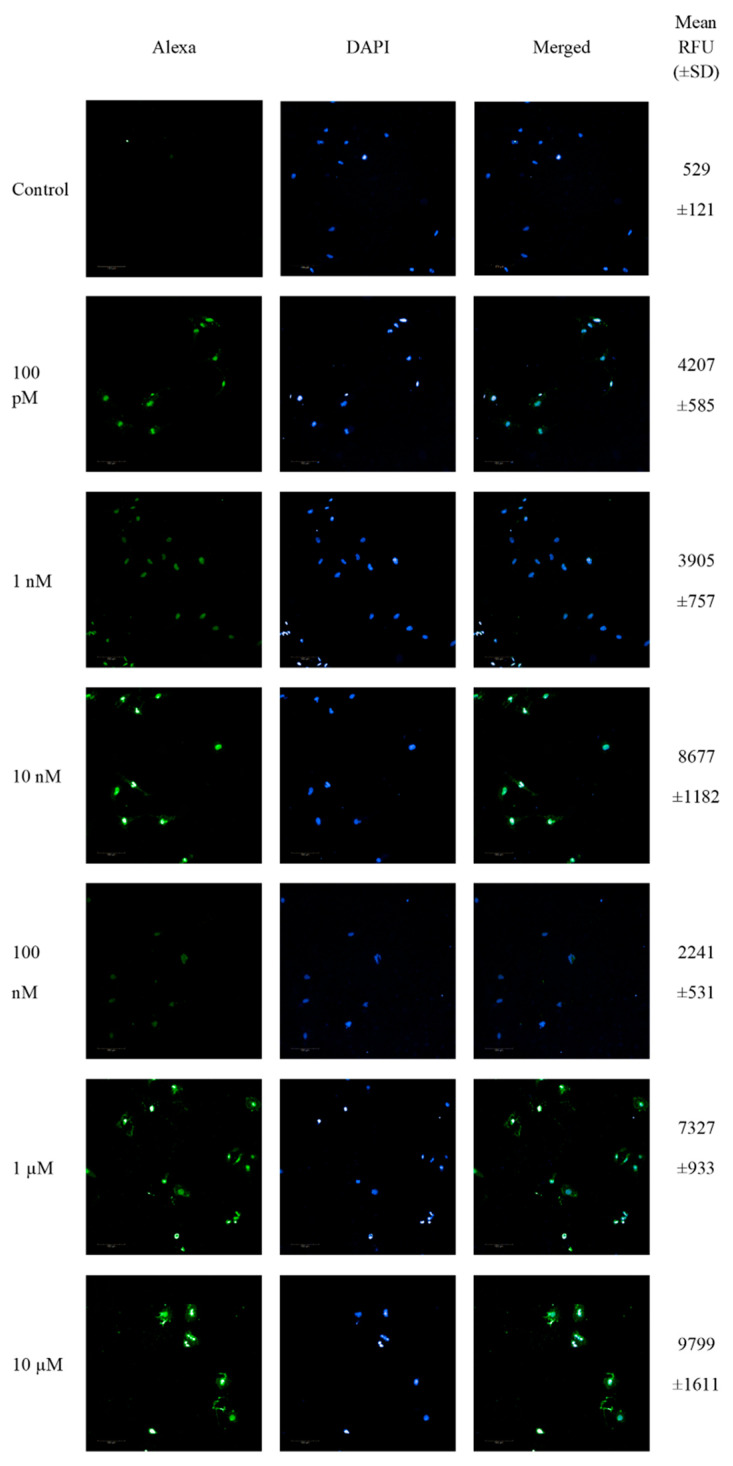
Confocal microscopy images of TUNEL assay. Detection of apoptotic cells based on DNA fragmentation labeling in the individual SW1088 cell line. FITC and DAPI staining for the nucleus. Representative images and the mean RFU values are shown.

SW1088 glioma cells were treated with 100 pM-10 µM 2-ME for 24 h, and then the number of cells showing DNA fragmentation features was examined. Representative pictures from confocal microscopy, as well as the mean RFU values, are presented in Figure 2.

**Figure 3:**
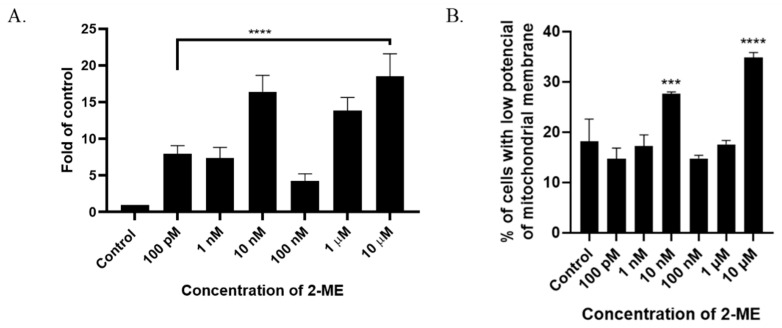
(**A**) 2-ME induces DNA strand breaks in SW1088 cells after treating cells with 100 pM–10 µM 2-ME. Values are the mean ± SE of three independent experiments, expressed as fold change compared to control cells. The data were analyzed using GraphPad Software, Inc., version 8, USA, by performing a one-way ANOVA; **** *p* < 0.0001 vs. control. (**B**) 2-ME decreases mitochondrial membrane potential in SW1088 cells. Values are the mean ± SE of three independent experiments. The data were analyzed using GraphPad Software, Inc., version 8, USA, by performing one-way ANOVA; *** *p* < 0.001, **** *p* < 0.0001 vs. control.

The analysis of TdT-labeled cells showed a statistically significant increase in the RFU of cells with DNA breaks compared to the control (non-treated cells). For SW1088 glioma cells, the RFU of TdT-labeled cells in the control (cells not treated with 2-ME) were considered 1, and for samples treated 24 h with 2-ME, the results were as follows: 100 pM—7.96 (±0.11); 1 nM—7.39 (±1.43); 10 nM—16.41 (±2.24); 100 nM—4.24 (±1.00); 1 µM—13.86 (±1.77); and 10 µM—18.54 (±3.05), relative to the control (Figure 3A).

### 3.5. 2-ME Decreases Mitochondrial Membrane Potential

A characteristic feature of the early stages of cell death is a disturbance in the functioning of mitochondria, manifested by a change in their membrane potential and redox potential. Nitro-oxidative stress is also associated with the depolarization of the mitochondrial membrane. In the next stage of the study, the mitochondrial potential of the cell membrane was analyzed after incubation for 24 h with 2-ME in a concentration range of 100 pM–10 µM. As a result, an increased number of cells with abnormally decreased mitochondrial potential was observed at 10 nM and 10 µM 2-ME, which was 27.63% (±0.38) and 34.80% (±1.04) in comparison to the control (18.13%, ±4.48) (Figure 3B).

### 3.6. 2-ME Induces Reactive Nitrogen Species (RNS) in SW1088 Glioma Cell Line

One possible mechanism for the cytotoxic and cytostatic actions of 2-ME is the induction of oxidative stress in treated cells. Taking into account the results of cytotoxicity, the intracellular levels of ROS were analyzed in the next stage of the study. The measurement was performed using the flow cytometry technique. Due to the low persistence of ROS, cells were incubated with 2-ME for 6 h [28].

**Figure 4:**
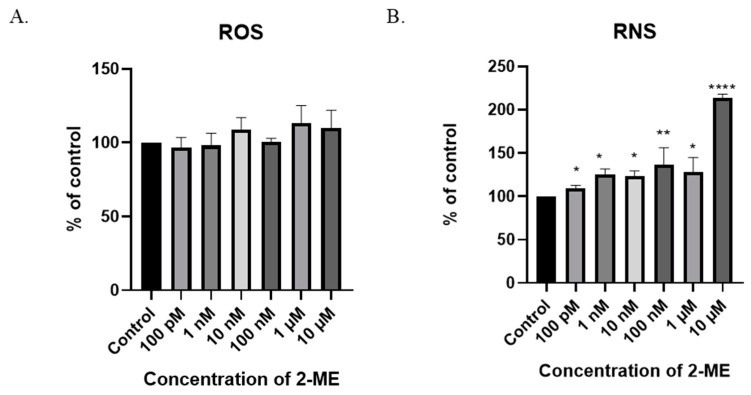
The induction of nitro-oxidative stress by 2-ME. (**A**) No significant change in intracellular ROS levels after treatment of SW1088 cells with 100 pM–10 µM 2-ME was observed. (**B**) Increase in intracellular RNS levels after treatment of SW1088 cells with 100 pM–10 µM 2-ME. Values are the mean ± SE of three independent experiments, expressed as % of control cells. The data were analyzed using GraphPad Software, Inc., version 8, USA, by performing one-way ANOVA; * *p* < 0.05, ** *p* < 0.01, **** *p* < 0.0001 versus control.

In the SW1088 glioblastoma cell line, none of the 2-ME concentrations used increased the ROS level at a statistically significant level (Figure 4A). Considering the above, the effect of 2-ME on the induction of nitro-oxidative stress in the treated cells was then examined by analyzing the intracellular level of RNS. The measurement was performed using the flow cytometry technique. Due to the low stability of RNS, cells were incubated with 2-ME for 6 h [28].

A six-hour treatment of SW1088 glioblastoma cells with 2-ME increased the level of RNS at all the concentrations used: 100 pM—110% (±3.16); 1 nM—125% (±6.47); 10 nM—123% (±6.2); 100 nM—136% (±20.0); 1 µM—128% (±16.6); and 10 µM—214% (±4.17) (Figure 4A).

NO is synthesized from L-arginine, nicotinamide adenine dinucleotide phosphate (NADPH), and oxygen by enzymes of the NOS family. As increased levels of NO have already been shown, the next stage of the research was to examine the level of three NOS isoforms: nNOS, iNOS, and eNOS.

**Figure 5:**
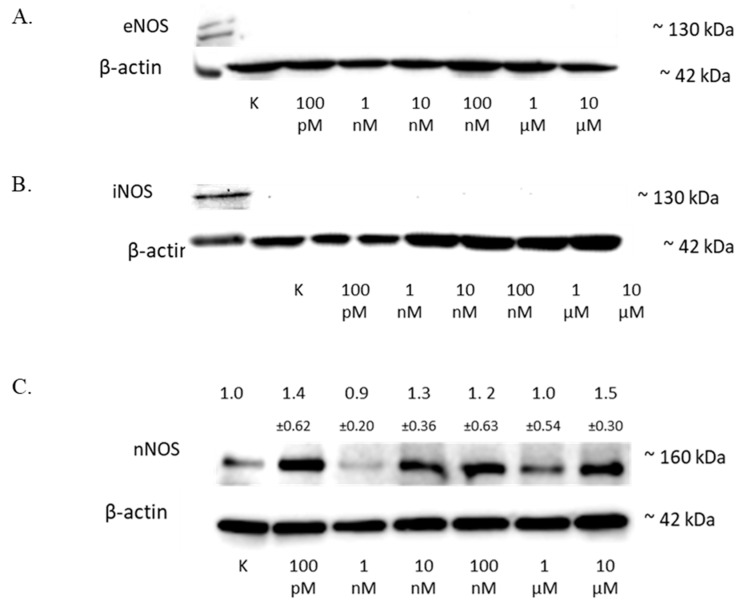
The influence of 2-ME on NOS in SW1088 cells. (**A**) No effect on eNOS levels in SW1088 glioma cell line treated with 100 pM–10 µM 2-ME assessed by Western blot analysis. (**B**) No effect on iNOS levels in SW1088 glioma cell line treated with 100 pM–10 µM 2-ME assessed by Western blot analysis. (**C**) The effect on nNOS levels in SW1088 glioma cell line treated with 100 pM–10 µM 2-ME was assessed by Western blot analysis. Densitometric analysis of the nNOS/β-actin ratio was performed using Quantity One 4.6.6 software. Representative immunoblots from one membrane are shown. Values are the mean ± SD of three independent experiments.

The treatment of the SW1088 cell line with 100 pM–10 µM 2-ME exhibited no effect on the level of eNOS (Figure 5A) or iNOS (Figure 5B) in the cells.

The analysis of the nNOS level by Western blot showed that 2-ME influenced the expression of this protein in cells (Figure 5C), and the results for the different concentrations were as follows: 100 pM—1.40 (±0.62); 1 nM—0.9 (±0.20); 10 nM—1.3 (±0.36); 100 nM—1.2 (±0.63); 1 µM—1.0 (±0.37); and 10 µM—1.5 (±0.30).

### 3.7. Regulation of HSP by 2-ME in SW1088 Glioma Cells

**Figure 6:**
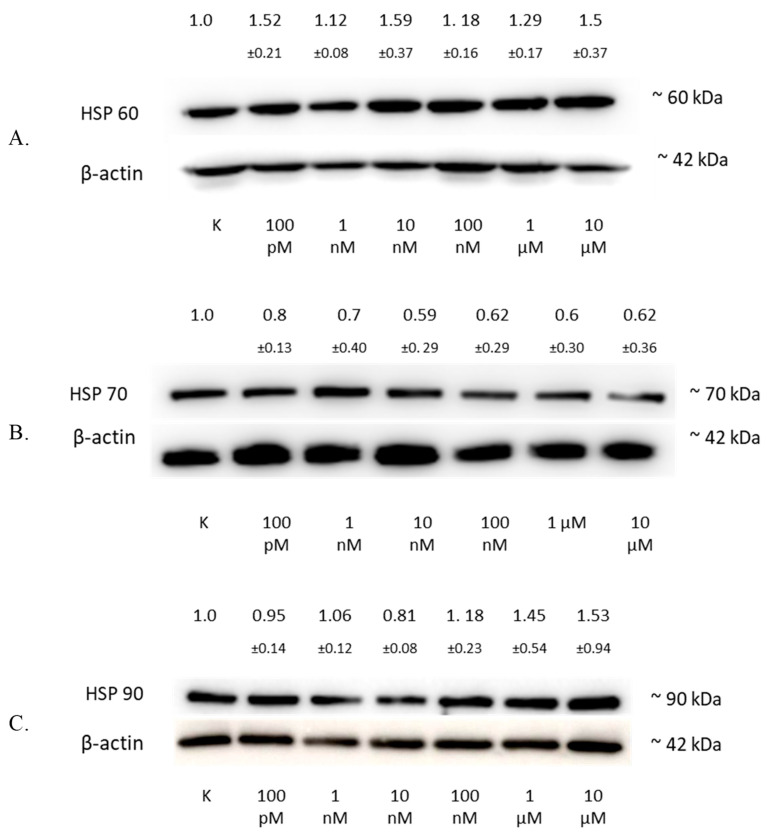
The influence of 2-ME on HSP in SW1088 cells. (**A**) The effect on HSP60 levels in the SW1088 glioma cell line treated with 100 pM–10 µM 2-ME was assessed by Western blot analysis. (**B**) The effect on HSP70 levels in the SW1088 glioma cell line treated with 100 pM–10 µM 2-ME was assessed by Western blot analysis. (**C**) The effect on HSP90 levels in the SW1088 glioma cell line treated with 100 pM–10 µM 2-ME was assessed by Western blot analysis. Densitometric analysis of HSP60/β-actin, HSP70/β-actin, and HSP90/β-actin ratios were performed using Quantity One 4.6.6 software. Representative immunoblots from one membrane are shown. Values are the mean ± SD of three independent experiments.

The analysis of the HSP60 protein level by Western blotting showed an increase in the expression of this protein in the SW1088 glioma line after 24 h of incubation with 2-ME, which were: 100 pM—1.52 (±0.21); 1 nM—1.12 (±0.08); 10 nM—1.59 (±0.37); 100 nM—1.18 (±0.16); 1 µM—1.29 (±0.17); and 10 µM—1.5 (±0.37), expressed as fold change compared to the control (Figure 6A).

The analysis of the HSP70 protein level by Western blotting showed a decrease in the expression of this protein in the SW1088 glioma line after 24 h of incubation with 2-ME, which were: 100 pM—0.80 (±0.13); 1 nM—0.70 (±0.40); 10 nM—0.59 (±0.29); 100 nM—0.62 (±0.29); 1 µM—0.60 (±0.30); and for 10 µM—0.62 (±0.36), expressed as fold change compared to the control (Figure 6B).

The analysis of the HSP90 protein level by Western blotting showed an increase in the expression of this protein in the SW1088 glioma line after 24 h of incubation with 2-ME, and the results were as follows: 1 nM—1.06 (±0.12); 100 nM—1.18 (±0.23); 1 µM—1.45 (±0.54); and 10 µM—1.53 (±0.94), expressed as fold change compared to the control. However, incubation with 100 pM and 10 nM 2-ME resulted in a decrease in HSP90 expression, which was 0.95 (±0.14) and 0.81 (±0.08), expressed as fold change compared to the control, respectively (Figure 6C).

## 4. Discussion

2-ME inhibits the proliferation of human glioblastoma cell lines. It induces apoptosis in SW1088 cells at both physiologically and pharmacologically relevant concentrations, whereas necrosis is observed at the highest pharmacological concentration. Moreover, we observed cell cycle blockage at all used concentrations. In recent years, a few studies have analyzed 2-ME in glioma cell culture [1]. Lis et al. [12] evaluated the impact of 2-ME on a human glioblastoma cell line in vitro. They used human GBM cell lines U138, U87, and T98 and compared them with rat astrocytes obtained from traumatized adult rat striata. The achieved results showed that all investigated GBM cell lines were vulnerable to 2-ME in pharmacologically relevant concentrations in a dose-dependent manner. The study demonstrated that the cells were blocked at the G2/M phase of the cell cycle, and apoptosis was induced. In parallel, Kumar et al. [29] published data consistent with the above [12], demonstrating that 2-ME greatly suppresses the development of cancer cells by primarily inhibiting cell cycle progression in the G2/M phase. The investigation of medulloblastoma (MB)—a primitive neuroectodermal tumor—cell lines (D341, DAOY, and D283) and two HGG cell lines, T-98-G and U-87MG, revealed that 2-ME treatment induces apoptosis via phosphorylation of cdc25C regulatory proteins and the activation of caspase 3. It is hypothesized that the inactivation of cdc2 proteins by hyperphosphorylation and interaction with 14-3-3 proteins prevents cells from entering the G2/M phase [30].

In the presented research model, concentrations corresponding to the physiological and pharmacological levels of 2-ME were used. After 24 h treatment of the SW1088 glioma line, a decrease in cell viability was observed only at 2-ME concentrations corresponding to the pharmacological ranges. However, the effect of 2-ME on the viability of three malignant human glioma cell lines (U87MG, U138MG, and LN405) and one malignant rat glioma cell line (RG-2) was investigated using the MTT assay. There was a noteworthy reduction in the number of viable cells in all cell lines [31]. In addition, Lis et al. showed a drop in the viability of U87, U138, and T98 glioblastoma cells after exposure to 2-ME [12].

In the experimental models used, we showed that the cytostatic mechanism of action of 2-ME is the arrest of cells in the subG1 phase, which proves the induction of apoptosis. 2-ME was also found to increase the cell number in the subG1 phase in hippocampal cells [23]. Additionally, physiologically relevant concentrations of 2-ME increase the number of SW1088 cells in the G2/M phase. The treatment of U87 glioma cells with 2-ME blocked cells in the G2/M phase of the cell cycle [12]. Cell cycle blockage in the G2/M phase by 2-ME corresponds to observations in other malignant cancers, including osteosarcoma [32], leukemia [33], and prostate cancer [34].

Apoptosis, also called programmed cell death, is a physiological process that controls the proper functioning of the body [35]. Deregulation of the apoptotic cell death pathway is a hallmark of tumors. Alterations in the apoptotic process are responsible not only for the formation and progression of cancer but also for therapy resistance. Most anticancer drugs currently used in clinical oncology use intact apoptotic signaling pathways to induce cancer cell death [36]. Our team was the first to demonstrate the induction of apoptosis by 2-ME in SW1088 glioma cells. It is known, however, that pharmacological concentrations of 2-ME induce caspase 3 in glioblastoma cells of the U87MG line, which indicates the process of apoptosis [31]. Numerous studies have proven that 2-ME causes apoptosis in other neoplasms, including, among others, melanoma cells [37], osteosarcoma [21], prostate [38,39], and breast cells [40].

The observed rise in the number of cells with lower mitochondrial potential in SW1088 glioma cells treated with 2-ME suggests that mitochondria may also be a target for 2-ME. Mitochondria are the center of energy metabolism via oxidative phosphorylation, and they also play crucial roles in cancer development and tumor anabolism [41]. As cancer cells, including glioma, were already reported to favor abnormal energy production by aerobic glycolysis, therapeutic attempts to target signaling or metabolic pathways have drawn much attention [42,43,44]. We formerly demonstrated that 2-ME decreases mitochondrial membrane potential in SH-SY5Y neuroblastoma cells [45] and inhibits mitochondrial biogenesis by regulating PGC-1α, COXI, and SIRT3 as a result of the nuclear recruitment of nNOS and NO generation in 143B osteosarcoma cells [26]. Complex I in mitochondria may affect the production of ROS, thereby meddling with the effectiveness of radiation. Increased mitochondrial metabolism can augment the oxygen requirement in tumor cells and develop a hypoxic microenvironment, thereby enhancing the radiation resistance of glioma cells. After the rotenone-mediated inhibition of complex I, U87MG glioblastoma cells were susceptible to X-ray radiation. However, the precise mechanism of mitochondrial metabolism in glioma radioresistance requires further investigation [46]. Mitophagy, a subtype of autophagy, is the principal mechanism for the deterioration of poorly functioning or damaged mitochondria. Mitophagy is necessary for maintaining cellular homeostasis, maintaining mitochondrial quality, and protecting cells from the deleterious effects of damaged mitochondria [47]. Interestingly, Huang et al. discovered that cannabidiol (CBD) inhibits the growth of multiple glioma cell lines, including A172, LN18, U87 MG, U251, U118 MG, and primary glial cells. The mechanism of action of CBD was the induction of mitochondrial damage and the lethal arrest of mitophagy, resulting in autophagic cell death [48]. In addition, 2-ME was also found to regulate mitochondrial dynamics in 143B osteosarcoma cells by stimulating mitochondrial fission and autophagy [49]. The subsequent upregulation of Drp1 and BAX proteins by 2-ME suggests the activation of the intrinsic apoptosis pathway [49]. Remarkably, Drp1 already mediates the activation of mitophagy, which may play a role in controlling mitochondrial content, mitochondrial divisions, metabolism, and apoptotic signaling in cancer cells [50]. As molecular pathways of cancer development and cancerogenesis may overlap [51,52], the role of mitochondria in both processes is very interesting. We believe that 2-ME may be a missing link connecting these two pathways [25].

DNA damage is a vital factor of cell vulnerability to chemotherapeutic drugs intended to eradicate tumor cells. In the presented study, the induction of DNA damage was observed after treatment with pharmacologically relevant concentrations of 2-ME. Moreover, DNA strand breaks induced by 2-ME were previously detected in the 143B osteosarcoma cell line [22] and mouse hippocampal cells HT-22 [23]. Additionally, 2-ME significantly enhanced radiation-induced genomic damage in T98G and U251MG cell lines [53]. Furthermore, we previously proved that RNS generated by 2-ME are inducers of DNA damage due to their affinity toward the guanine base of DNA [54].

The induction of oxidative stress is a common factor leading to the development of both neurodegeneration and cancers [25,55,56], and one of the mechanisms of 2-ME is the generation of ROS and RNS in many cell lines [21,22,23,45,54,57,58,59,60]. The mode of action of many anticancer agents is the induction of oxidative stress. For glioblastoma multiforme (GMB), three key signaling pathways have been recognized as the most unregulated in glioblastoma, notably RTK/Ras/phosphoinositide 3-kinase (PI3K) pathway activation and the suppression of the p53 protein and retinoblastoma protein (Rb) [61]. Kinetin riboside (KR) and newly created derivatives (8-azaKR and 7-deazaKR) interfere with the redox state of cancer cells by selectively affecting molecular pathways essential for cell proliferation. KR and 7-deazaKR are efficient anticancer drugs and could be attractive options for oxidative therapy, concentrating on the cellular redox conditions of GBM cells and the inducement of apoptosis, according to previous findings [62]. The SW1088 glioma line showed no ROS induction despite incubation with 2-ME; however, it induced RNS across the entire concentration range of the compound at both physiologically and pharmacologically relevant concentrations, and in addition, it upregulated nNOS expression. Furthermore, we previously demonstrated that 2-ME induces a pro-apoptotic signaling cascade by augmenting cellular RNS synthesis [54]. The lack of ROS induction is a result of rapid O_2_^•−^ combination with ^•^NO to produce ONOO^−^ [54,63]. The possible physiological generation of ONOO−/ONOOH easily occurs in the case of certain pathophysiological conditions, such as cancer. Next, HOONO reacts with either ^•^NO or O_2_^•−^ to generate ^•^NO_2_ [54,64]. Together, ONOO− and ^•^NO_2_ are known to promote both apoptotic and necrotic cell death pathways [63].

In the search for the mechanism of action of 2-ME, we turned our attention to HSPs, which have a cytoprotective effect, including in cancer cells, and their overexpression has been demonstrated in many cancers [65,66,67,68,69,70,71]. Interestingly, some HSPs are believed to be predictive biomarkers for human brain glioma [72].

Cancer cells actively release HSP60, which promotes angiogenesis. HSP60 seems to have a cytoprotective effect on cellular stressors and may also increase the anti-apoptotic effect. HSP60 may serve as a biomarker for the prognosis and diagnosis of cancer [68,69,73]. 2-ME increased HSP60 expression in SW1088 glioma cells at all of the concentrations used. HSP60 activity has been previously demonstrated in glioblastoma [65], and the results reveal that HSP60 silencing inhibits glioma progression via deactivation of the mTOR pathway, suggesting that HSP60 is a possible therapeutic target for the treatment of glioblastoma [74]. Therefore, it is necessary to study the influence of 2-ME on the activity of the mTOR pathway in neuronal cells. It was previously proven that 2-ME inhibits the PI3K/Akt/mTOR pathway in skin fibroblasts [75].

When HSP70 levels remain elevated, they play a crucial role in tumor progression by encouraging carcinogenesis. It is considered a survival factor due to its expression in tumors and its anti-apoptotic activity [76,77,78]. HSP70 suppresses apoptosis by binding to Bax and preventing its translocation to the mitochondria [79]. In the presented research, the decreased level of the HSP70 protein under the influence of 2-ME in SW1088 glioblastoma cells was observed. Intriguingly, 2-ME did not affect HSP70 expression in 143B osteosarcoma cells [80] or in A375 melanoma cells [37]. HSP70 was shown to prevent the aggregation of oxidized glyceraldehyde-3-phosphate dehydrogenase (GAPDH) and to diminish cell death induced by hypoxia [81]. The overexpression of HSP70 supports the ubiquitination and proteasomal degradation of nNOS [82], while its inhibition sensitizes cancer cells to apoptosis [83]. Delaying the function of Hsp70 and its interaction with GAPDH may contribute to rendering tumors receptive to chemotherapy and resistant to a variety of environmental stressors [81].

HSP90 levels are also elevated in cancer cells. HSP90 plays an essential role in facilitating neoplastic transformation and thus is crucial for the development of solid malignancies. Hsp90 activity in cancer cells appears to be closely related to the overall proliferation potential of these malignant cells and has been shown to allow cancer cells to avoid apoptotic death [66,84]. In addition, HSP90 can function as an allosteric regulator of NOS isoenzymes, encouraging the acquisition of the active conformation or boosting NOS’ affinity for the Ca2+/calmodulin sensor. Averna et al. demonstrated that the susceptibility of nNOS to the proteolytic effect of calpain is substantially lowered in the presence of equimolar amounts of HSP90, suggesting a new mechanism involving the combination of NOS and HSP90 and the concurrent recruitment of active calpain in ternary complexes in which the proteolysis of both NOS and HSP90 isoenzymes is considerably decreased [85]. In the presented research, an increased level of the HSP90 protein was observed after the incubation of SW1088 glioblastoma cells with 2-ME. An elevated level of HSP90 as a result of 2-ME activity was previously found in the 143B osteosarcoma cell line [80]. Moreover, Hsp90 takes part in 2-ME-mediated nNOS nuclear translocation, resulting in cancer cell death [80].

## 5. Conclusions

2-ME induces apoptosis in SW1088 glioma cells and alters its functions by generating RNS. The induction of RNS causes DNA damage, which culminates in the death of cancer cells. Numerous prior investigations have revealed that 2-ME has anticancer and antiangiogenic properties [14,15], but the mechanism of action was still unclear. Our team revealed that the genotoxic activity of 2-ME is a result of RNS’ affinity to the guanine base of DNA [54]. Here, for the first time, we demonstrated the induction of nitro-oxidative stress in a glioblastoma grade III cellular model by 2-ME with a correlation with nNOS and HSP activity. Moreover, we established that mitochondria in both SW1088 glioma cells and 143b osteosarcoma cells are a target for 2-ME [26,49]. For more reliable conclusions, there is a special need for in vivo investigation of ROS and RNS blood levels, as well as estrogen derivatives, in patients suffering from glioma cancer. Furthermore, 2-ME was recently found to induce S-palmitoylation in A549 lung cancer cells [86], so this highlights palmitoylation as a clinically valid, innovative target of 2-ME for anticancer therapy for further investigation.

## Data Availability

Not applicable.
